# Self-Efficacy and Attachment Styles Mediate the Impact of Early Life Trauma on Negative Emotional States in Adults Living in Portugal

**DOI:** 10.1007/s40653-025-00812-z

**Published:** 2026-01-08

**Authors:** Catarina Gomes Coelho, Luíza Cerri, Jorge Leite, Paulo P. P. Machado, Sandra Carvalho

**Affiliations:** 1 Psychotherapy and Psychopathology Research Lab – CIPsi, School of Psychology, University of Minho, Braga, Portugal; 2 Psychological Neuroscience Laboratory, Centro de Investigação em Psicologia (CIPsi), Department of Basic Psychology, School of Psychology, University of Minho, Gualtar, Braga, Portugal, Campus de Gualtar, Rua da Universidade, Braga, Gualtar 4710–057 Portugal; 3 CINTESIS@RISE, CINTESIS.UPT, Portucalense University, Porto, Portugal

**Keywords:** Early life trauma, Negative emotional states, Self-efficacy, Anxious attachment style, Avoidant attachment style

## Abstract

While early life trauma (ELT) is known to contribute to poor physical and psychological health in adulthood, not all individuals exposed to ELT develop mental illness. This suggests the presence of potential mediating factors that influence this relationship. This study examines the mediating effects of self-efficacy and attachment styles on the relationship between ELT and negative emotional states (NEs) in a large cohort of people in Portugal. A total of 936 participants completed standardized psychological assessments via an online survey. Structural equation modeling (SEM) and mediation analysis were employed to examine the relationships among ELT, self-efficacy, attachment styles, and NEs. The findings indicated a strong correlation between ELT and diminished self-efficacy, as well as elevated levels of anxious and avoidant attachment patterns. These factors mediated the link between ELT and adverse emotional states. The results underscore the significant function of self-efficacy and attachment patterns as primary mediators in the emotional outcomes of ELT. Addressing these aspects via trauma-informed therapies may alleviate the psychological cost of ELT. Future longitudinal research are necessary to establish causal relationships and further examine these pathways.

**Supplementary Information:**

The online version contains supplementary material available at https://doi.org/10.1007/s40653-025-00812-z.

## Introduction

The effects of early life trauma (ELT) are extensively established, demonstrating substantial associations with various mental health illnesses and adverse emotional states (NEs) in adulthood. Early Life Trauma (ELT), encompassing experiences such as abuse, neglect, and familial dysfunction, can result in enduring psychological repercussions (Kessler et al., 2010). The relationship between ELT and adult emotional issues is well documented, although the fundamental mechanisms driving this connection remain ambiguous. Recent studies indicate that self-efficacy and attachment styles may modulate the impact of ELT on NEs, however substantial evidence about these mechanisms remains scarce (Bockting et al., 2024; Corcoran & McNulty, 2018). Comprehending these mediating aspects within various cultural contexts is crucial for formulating successful, culturally attuned trauma-focused therapies.

Portugal, characterized by strong family bonds and collectivist tendencies, places significant emphasis on familial relationships, which can both buffer and exacerbate the effects of ELT. Cultural norms might affect the formation of insecure attachment patterns, since emotional repression in families may result in avoidant or anxious attachment styles in adulthood (Pinto et al., 2021). Moreover, cultural norms about resilience and emotional regulation may influence self-efficacy, as individuals from emotionally constrictive situations are less inclined to cultivate a robust sense of personal agency (Silva et al., 2023). The stigma associated with mental health in Portugal may exacerbate the internalization of negative emotions, hence aggravating the psychological consequences of ELT (Aluh et al., 2022). Identifying these cultural characteristics is crucial for comprehending the study’s findings and formulating culturally appropriate, trauma-informed therapies for the Portuguese community.

Self-efficacy, defined as an individual’s belief in their capacity to undertake specified tasks and their assurance in effectively executing these tasks, was proposed by Bandura in his foundational work on social cognition theory. This concept underscores the significance of self-efficacy in influencing individuals’ emotions, cognition, and behavior across diverse contexts (Bandura, 1997). Studies indicate that persons possessing elevated self-efficacy have enhanced regulation of their emotions, behaviors, and decision-making, hence fostering general psychological well-being (Komarraju et al., 2014). Consequently, self-efficacy is pivotal in stress management, affecting individuals’ assessment of stressors and the coping methods they select and employ (Villada et al., 2018).

Previous research indicates that exposure to ELT can adversely affect sociopsychological resources like as self-efficacy (Weindl et al., 2018). Child maltreatment is believed to hinder the development of self-efficacy by restricting opportunities for exploration and personal growth (Cohrdes & Mauz, 2020; Ryan et al., 1996). Studies indicate that young people who encountered elevated degrees of emotional neglect and sexual abuse have diminished self-efficacy and a lower quality of life (Cohrdes & Mauz, 2020). Additionally, Zhang et al. (2023), found a significant relationship between childhood abuse and mental illness, with self-efficacy mediating this relationship. Furthermore, subjective social status mediated this mediating pathway. The authors suggested that enhancing self-efficacy may prevent or reduce depressive symptoms, particularly among university students who have experienced ELT. Another study examining the relationship between early family violence and the development of post-traumatic stress symptoms (PTSS) in young adulthood found that lower self-efficacy was associated with both variables. Self-efficacy also mediated the link between parental violence and PTSS (Haj-Yahia et al., 2019).

Attachment style is also recognized as an important mediator of the relationship between ELT and NEs (Degnan et al., 2022; Muller et al., 2012). Attachment styles. Which define how individuals relate to others in intimate and caregiving relationships, are formed early in life and often persist into adulthood (Donisi et al., 2022). Two main dimensions of insecure attachment are widely acknowledged: anxious and avoidant. Anxious attachment is characterized by heightened activation of the attachment system, leading to a constant need for support and reassurance. In contrast, avoidant attachment involves a deactivated attachment system, resulting in the suppression of psychological needs, self-reliance, and emotional distance from others (Liu & Ma, 2019). Individuals with higher levels of anxious attachment often regulate their emotions through heightened emotional responses, maintaining high levels of negative emotions (Clear et al., 2020; Mikulincer et al., 2003). Conversely, individuals with high levels of avoidant attachment struggle to form close, emotionally involved relationships. They tend to suppress negative thoughts, repress negative memories, project negative self-perceptions onto others, and deny basic fears, making it difficult to acknowledge negative emotions (Kobak et al., 1993; Simon et al., 2019).

Early traumatic events are associated with the formation of insecure attachment patterns and internal working models that impair the effective control of emotions (Fuchshuber et al., 2019). Anxious and avoidant attachment styles are frequently linked to psychopathologies, including depression and anxiety (Bosmans et al., 2010; Cantazaro & Wei, 2010; Gormley & McNiel, 2010). Corcoran and McNulty (2018) discovered that the association between ELT and psychological discomfort was mediated by specific attachment dimensions, with anxious attachment in general relationships serving as the most significant mediator. A separate study indicated that anxious-insecure attachment style and personal resilience resources mediate the association between ELT and psychopathological symptoms, while avoidant-insecure attachment correlates with diminished interpersonal resilience, which moderates the link between recent trauma experiences and psychopathological symptoms (Rossi et al., 2023). Furthermore, Ye and colleagues (2024) shown that insecure attachment patterns and maladaptive emotion regulation mechanisms substantially influence the correlation between unpleasant childhood events and depressive symptoms in adulthood.

Previous studies have identified connections among self-efficacy, attachment patterns, early life trauma (ELT), and negative emotional states (NES), indicating that these elements may affect their interplay within particular cultural contexts. Nonetheless, a considerable gap persists in the literature about the mediating influence of self-efficacy and attachment patterns in this relationship.

Most studies have not thoroughly examined how these variables function as mediators, particularly across diverse cultural backgrounds, and even fewer studies have focused on the Portuguese population. These studies predominantly concentrated on the direct relationship between ELT and NEs, quality of life, attachment styles, and the role of potential mediators and moderators in the dynamic remains overlooked (Aluh et al., 2022; Pinto et al., 2021; Silva et al., 2023). This underscores the need for further investigation to enhance our understanding of these pathways and their implications for developing culturally sensitive, trauma-informed interventions.

### The Current Study

The main objective of this research is to assess the mediating role of self-efficacy and attachment styles in the relationship between ELT and adulthood NEs, as assessed by the DASS-21, within a cohort of population living in Portugal. The secondary objectives are 1) to analyze differences between individuals with or without ELT experiences concerning: (a) sociodemographic and clinical characteristics; (b) the development of NEs (anxiety, depression, and stress); (c) self-efficacy perceptions; and (d) prevalence of anxious and avoidant attachment styles; and 2) to examine the relationships between ELT, the development of adulthood psychological symptoms, attachment styles, and self-efficacy.

**Fig. 1:**
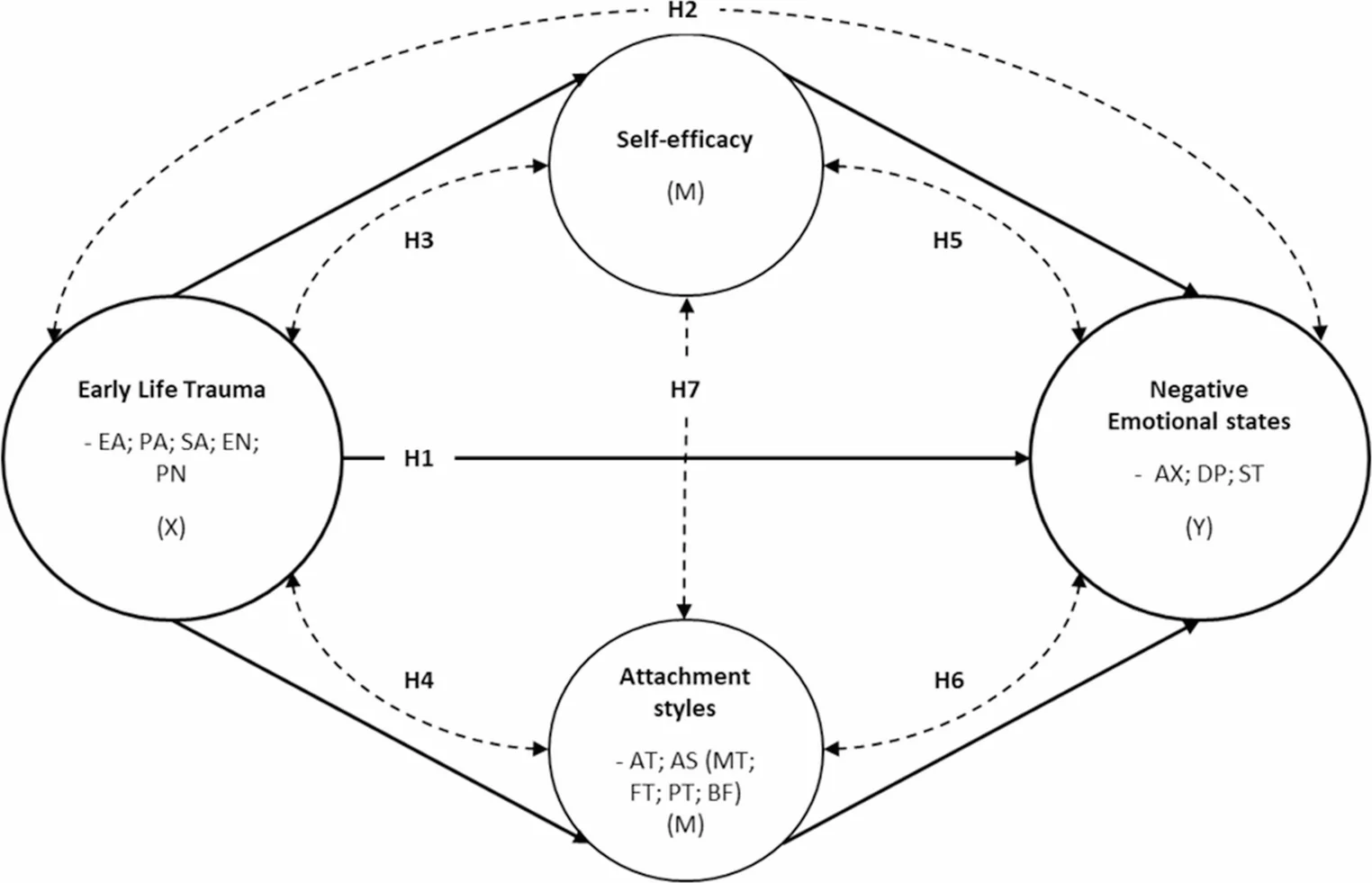
Hypothesis Research Model. **Note.**AN, Anxiety; AS, Anxious; AT, Avoidant; BF, Best Friend; EA, Emotional Abuse; DP, Depression; EN, Emotional Neglect; FT, Father; MT, Mother; PA, Physical Abuse; PN, Physical Neglect; PT, Partner; ST, Stress

Based on these objectives, the hypothesized research model posits that self-efficacy and attachment styles mediate the relationship between ELT and NEs (H1). Additionally, it is hypothesized that individuals with ELT (those with abuse and neglect childhood experiences) will exhibit: higher levels of anxiety, depression, and stress (H2); more anxious and avoidant attachment styles (H4); and, conversely, lowered self-efficacy scores (H3). Moreover, it is also expected that individuals with higher self-efficacy perception will report fewer NEs (H5); while those with more anxious and avoidant attachment styles will exhibit more NEs (H6). Finally, an association between self-efficacy perception and both anxious and avoidant attachment styles is also hypothesized (H7) (Fig. 1).

#### Method

##### Procedure and Participants

A cross-sectional study design was employed for the online survey conducted between September 2021 to March 2023. Data collection followed a snowball sampling technique, utilizing leaflets, social media posts, and participation links sent to various institutions in Portugal. Participants completed a set of psychological self-report questionnaires, including the Childhood Trauma Questionnaire, Depression Anxiety Stress Scales, General Self-Efficacy Scale, and Experiences in Close Relationships-Relationship Structures (among others). Prior to beginning the survey, all participants provided informed consent and were informed about the anonymity and confidentiality of their responses, as well as their right to withdraw from the survey at any time. Data were collected from 946 volunteers residing in Portugal. After applying the inclusion criteria (≧ 18 years old; fluent in Portuguese), 10 participants were excluded - one for not providing informed consent and nine for being under 18 years old.

The study was approved by the Ethics Committee for Research in Social and Human Sciences (approval ID: CEICSH 087/2021). The data presented in this study are part of a broader investigation that includes additional instruments.

## Measures and Instruments

### Sociodemographic and Clinical Characteristics Questionnaire

The Sociodemographic and Clinical Characteristics questionnaire consisted of 28 detailed inquiries aimed at collecting extensive information regarding participants’ personal and medical histories. The inquiries encompassed several subjects, including gender, age, nationality, educational qualifications, marital status, profession, and lifestyle elements such as substance consumption. The questionnaire also examined participants’ medical histories, encompassing both personal and familial health issues, to offer a comprehensive insight into their sociodemographic and clinical profiles.

## Early Life Trauma

The Childhood Trauma Questionnaire-Short Form (CTQ-SF) is a 28-item self-report instrument designed to assess five types of early life trauma: emotional, physical, and sexual abuse, as well as emotional and physical neglect. In addition, three items assess the validity of the answers using the minimization/denial subscale (Bernstein et al., 2003). Participants respond to items on a Likert scale ranging from 1 (never true) to 5 (very often true). Thus, participants were asked to retrospectively report experiences of early life trauma occurring during their childhood and adolescence. The scale has demonstrated adequate internal consistency in previous studies (*α* = 0.84) (Thombs et al., 2009). In this study, the CTQ-SF also evidenced adequate internal consistency, with a total scale reliability of *α* = 0.94 and subscales reliabilities as follows: emotional abuse (*α* = 0.87), physical abuse (*α* = 0.88), sexual abuse (*α* = 0.90), emotional neglect (*α* = 0.89), and physical neglect (*α* = 0.75). The cutoff values used in the current study were the ones that were used in Glaesmer et al. (2016) and Bernstein et al. (2003).

### Negative Emotional States

The Depression Anxiety Stress Scales (DASS) consists of 21 self-report items designed to assess the levels of anxiety, depression, and stress symptoms, within the past week. Responses are recorded on a Likert scale ranging from 0 (did not apply to me at all) to 3 (applied to me very much or most of the time). Previous research has shown the total scale and its three subscales to have adequate internal consistency, ranging from α = 0.82 to α = 0.93 (Henry & Crawford, 2005). In the current study, the scale also presented a good internal consistency, both in the total scale (*α* = 0.96) and in the subscales (anxiety: *α* = 0.91; depression: *α* = 0.93; stress: *α* = 0.91).

## Self-Efficacy

The General Self-Efficacy Scale (GSE) is a 10-item self-report instrument designed to assess participant ‘current perceived ability to cope with challenges, reflecting their present self-efficacy beliefs. Responses are rated on a Likert scale ranging from 1 (not at all true) to 4 (exactly true). The GSE has demonstrated adequate psychometric properties in previous research (*α* =.76) (Scholz et al., 2002). In this study, the scale showed a good internal consistency (*α* =.90).

## Attachment Styles

The Experiences in Close Relationships – Relationship Structures (ECR-RS) is a self-report instrument designed to assess current attachment patterns across various close relationships, including mother/mother figure; father/father figure; partner; and best friend, reflecting their present attachment tendencies. For each relationship, nine items are used, with three assessing avoidant attachment and six assessing anxious attachment. Responses are rated on a Likert scale from 1 (not at all true) to 7 (completely true). This instrument has demonstrated adequate internal consistency, with Cronbach’s Alpha values ranging from 0.83 to 0.87 for anxiety and from 0.81 to 0.92. for avoidance scales across the relationship domains (Moreira et al., 2015). In the present study, the internal consistency was good, in the total subscales (anxiety: *α* = 0.91; avoidance: *α* = 0.92) and four attachment figures with anxiety and avoidance scales for each one (Mother: anxiety - *α* = 0.87 and avoidance - *α* = 0.88; Father: anxiety - *α* = 0.88 and avoidance - *α* = 0.87; Partner: anxiety - *α* = 0.92 and avoidance - *α* = 0.86; Best friend: anxiety - *α* = 0.92 and avoidance - *α* = 0.87).

### Data Analysis

To account for the potential common method bias (CMB) due to the self-report nature of the data, Harman’s one-factor test was conducted using IBM SPSS Statistics 23. Since the total variance extracted by a single factor was 39.53%, which is below the recommended threshold of 50% (Aguirre-Urreta & Hu, 2019), CMB was not considered an issue in this study.

Additionally, Jeffrey’s Amazing Statistics Program (JASP) version 0.18.2.0 was used to evaluate convergent validity using the average variance extracted (AVE), discriminant validity with heterotrait-monotrait (HTMT) ratio, and reliability with Cronbach alpha (α) and Omega (𝜔) coefficients for the CTQ total scale and subscales. All AVE scores exceeded 0.40, indicating adequate validity (Dewi & Indra, 2024; Fornell & Larcker, 1981). Moreover, all HTMT ratio results were lower than 0.90, demonstrating adequate validity (Henseler et al., 2014). Furthermore, the α coefficients showed good results, ranging between 0.75 and 0.94, as well as the 𝜔 coefficients, which demonstrated results ranging between 0.79 to 0.92(Stensen et al., 2022) (for details, see Supplementary Information Table SI). One-way ANOVA and chi-square tests were conducted to examine the differences between the four groups, the control group (without or with minimal ELT experiences), and three groups with slight to extreme ELT experiences, regarding quantitative variables, sociodemographic and clinical characteristics. Afterward, bivariate correlation analyses were carried out using Pearson’s correlation coefficient, between ELT, psychological symptoms, self-efficacy, and attachment styles, with the software R studio. The mediation hypothesis was tested using structural equation modeling (SEM), including the two-step approach indicated by other studies (Anderson & Gerbing, 1988).

First, a second-order confirmatory factor analysis (CFA) of the CTQ-SF was performed. Next, a structural equation model was tested, using IBM SPSS AMOS 29 with the maximum likelihood method (Kline, 2016). In this model, the independent variable was the CTQ total score, the dependent variable was the DASS total score, and the mediating variables were GSE and anxiety and avoidant ECR-RS total scales. Several goodness of fit measures were used to evaluate how well the hypothesized model fit the observed results: *X*^*2*^/df, Root Mean Square Error of Approximation (RMSEA), Comparative Fit Index (CFI), and Tucker-Lewis Index (TLI). Usually, the model is considered to show good fit for RMSEA values below 0.06 and CFI and TLI values over 0.90 (Brown, 2006). Despite its sensitivity to sample size (Byrne, 2011), while considered to be less informative, the chi-square statistic was also reported.

Finally, simple mediation analyses were conducted using Process Model 4 to test whether self-efficacy, anxious attachment, and avoidant attachment significantly mediated the relationship between specific types of ELT and current psychological symptoms (anxiety, depression, and stress). A bootstrapping procedure with 5000 samples and a 95% confidence interval was used (Alfons et al., 2021), and beta values below 0.10 were considered non-significant.

## Results

### Sociodemographic Data

This research included 936 participants (age: *M* = 29.4; *SD* = 24; 75% female); of these, about 88% have Portuguese nationality, 76% of participants weren’t in a relationship, 65% completed a higher education, 18% reported psychiatric illness and 52% had direct family members with reported psychological problems. Regarding the ELT experiences, 67% of participants referred to ELT experiences (around 28% slight to moderate, 16% moderate to severe, and 23% severe to extreme).

Table 1 shows the sociodemographic variables, comparing the four groups (control group and groups with ELT experiences, slight to extreme). The results showed significant differences in all variables (*p* <.01; *p* <.001). The ELT groups displayed: higher age, which increases with ELT experience severity. While the female gender, Portuguese nationality, single marital status, and higher education were the most common characteristics across the four groups, the severe to extreme ELT group exhibited a higher number of male participants, a greater diversity of nationalities, more individuals who were cohabiting, married, or divorced, and a lower proportion of participants with higher education compared to the other groups. Regarding substance use, participants in the several to extreme ELT group related a higher frequency of alcohol and other substance consumption. Furthermore, the same group presented a higher prevalence of psychiatric illness and family psychological problems.

**Table 1:** Sociodemographic characteristics

	Control group (*n* = 308)	Slight to moderate ELT (*n* = 264)	Moderate to severe ELT (*n* = 151)	Severe to extreme ELT (*n* = 213)	df	χ2/F	*p*	Total group (*n* = 936)
	M (SD)/ *n* (%)	M (SD)/ *n* (%)	M (SD)/ *n* (%)	M (SD)/ *n* (%)				M (SD)/ *n* (%)
Age	27.5 (10.3)	28.3 (11.7)	29.3 (10.2)	33.4 (13.2)	3	12.7	*p* <.001	29.4 (11.6)
Gender					9	22.6	0.007	
Female	243 (78.9)	211 (79.9)	110 (72.8)	139 (65.3)				703 (75.1)
Male	64 (20.8)	53 (20.1)	41 (27.2)	71 (33.3)				229 (24.5)
Other	1 (0.3)	-	-	3 (1.4)				4 (0.4)
Nationality					6	41.9	*p* <.001	
Portuguese	285 (92.5)	249 (94.3)	127 (84.1)	165 (77.5)				826 (88.2)
Other nationalities	20 (6.5)	14 (5.3)	20 (13.2)	42 (19.7)				96 (10.3)
Dual nationality	3 (1.0)	1 (0.4)	4 (2.6)	6 (2.8)				14 (1.5)
Marital status					12	61.2	*p* <.001	
Single	249 (80.8)	204 (77.3)	106 (70.2)	117 (54.9)				676 (72.2)
Cohabiting	15 (4.9)	9 (3.4)	9 (6.0)	25 (11.7)				58 (6.2)
Married	39 (12.7)	42 (15.9)	30 (19.9)	50 (23.5)				161 (17.2)
Divorced	4 (1.3)	7 (2.7)	6 (4.0)	20 (9.4)				37 (4.0)
Widowed	1 (0.3)	2 (0.8)	-	1 (0.5)				4 (0.4)
Education					9	78.7	*p* <.001	
Elementary school	1 (0.3)	1 (0.4)	-	2 (0.9)				
Middle school	4 (1.3)	1 (0.4)	2 (1.3)	29 (13.6)				
High school	90 (29.2)	84 (31.8)	44 (29.1)	71 (33.3)				289 (30.9)
Higher education	213 (69.2)	178 (67.4)	105 (69.5)	111 (52.1)				
Alcohol use					3	17.6	0.001	
Yes	40 (13.0)	41 (15.5)	28 (18.5)	57 (26.8)				166 (17.7)
No	268 (87.0)	223 (84.5)	123 (81.5)	156 (73.2)				770 (82.3)
Other substances					3	36.9	*p* <.001	
Yes	5 (1.6)	8 (3.0)	6 (4.0)	27 (12.7)				46 (4.9)
No	303 (98.4)	256 (97.0)	145 (96.0)	186 (87.3)				890 (95.1)
Psychiatric illness					3	82.4	*p* <.001	
Yes	25 (8.1)	38 (14.4)	26 (17.2)	82 (38.5)				171 (18.3)
No	283 (91.9)	226 (85.6)	125 (82.8)	131 (61.5)				765 (81.7)
Family psychological problems					6	49.6	*p* <.001	
Yes	128 (41.6)	131 (49.6)	88 (58.3)	135 (63.38)				482 (51.5)
No	150 (48.7)	101 (38.3)	40 (26.5)	47 (22.1)				341 (36.4)
I don’t know	27 (8.8)	32 (12.1)	23 (15.2)	31 (14.6)				113 (12.1)

### Early Life Trauma Experiences End Clinical Data

Table 2 shows the prevalence of ELT experiences and the differences between the four groups regarding clinical characteristics. Concerning the ELT groups, around 42% of the participants reported emotional abuse, 19% physical abuse, 14% sexual abuse, 25% emotional neglect and 29% physical neglect. Post-hoc Bonferroni tests showed that these groups (for general ELT and specific ELT experiences) presented higher scores of general and specific psychological symptoms (anxiety, depression, and stress), anxiety, and avoidant attachment styles compared with the control group (*p* <.05). Additionally, the control group presented higher results of self-efficacy, compared with the ELT groups (for general ELT and specific ELT experiences) (*p* <.05).

**Table 2:** Early Life Trauma and Clinical characteristics

		None to minimal	Slight to moderate^(a)^	Moderate to severe^(b)^	Severe to extreme^(c)^	df	χ2/F	*p*
Emotional abuse	Cutoff	5–8	9–12	13–15	16–25	3	3391.3	*p* <.001
	n (%)	489 (52.2)	226 (24.1)	99 (10.6)	122 (13.0)			
	*M* (*SD*)	6.1 (1.1)	10.5 (1.1)	13.9 (0.8)	18.6 (2.5)			
Physical abuse	Cutoff	5–7	8–9	10–12	13–25	3	4134.8	*p* <.001
	n (%)	760 (81.2)	45 (4.8)	74 (7.9)	57 (6.1)			
	*M* (*SD*)	5.2 (0.5)	8.3 (0.5)	10.8 (0.5)	15.4 (2.3)			
Sexual abuse	Cutoff	5	6–7	8–12	13–25	3	3151.4	*p* <.001
	n (%)	710 (75.9)	95 (10.1)	78 (8.3)	53 (5.7)			
	*M* (*SD*)	5 (0.0)	6.5 (0.5)	9.2 (1.3)	16.6 (3.4)			
Emotional neglect	Cutoff	5–9	10–14	15–17	18–25	3	3303.1	*p* <.001
	n (%)	430 (45.9)	270 (28.8)	139.0 (14.9)	97 (10.4)			
	*M* (*SD*)	6.7 (1.4)	11.8 (1.4)	15.9 (0.77)	19.9 (1.9)			
Physical neglect	Cutoff	5–7	8–9	10–12	13–25	3	3771.3	*p* <.001
	n (%)	666 (71.2)	119 (12.7)	71 (7.6)	80 (8.5)			
	*M* (*SD*)	5.4 (0.69)	8.6 (0.50)	10.9 (0.87)	15.3 (2.0)			
**Anxiety**								
Early Life Trauma	*M* (*SD*)	3.7 (4.4) ^a***, b***, c***^	5.6 (5.3) ^c***^	5.7 (5.2) ^c***^	9.1 (5.6)	3	48.5	*p* <.001
Emotional abuse	*M* (*SD*)	4.0 (4.5) ^a***, b***, c***^	6.7 (5.6) ^c***^	7.4 (5.3) ^c***^	10.2 (5.5)	3	57.5	*p* <.001
Physical abuse	*M* (*SD*)	5.1 (5.2) ^b***, c***^	6.7 (5.3) ^c***^	7.9 (5.8) ^c**^	11.5 (5)	3	31.9	*p* <.001
Sexual abuse	*M* (*SD*)	5.1 (5.2) ^a*, b***, c***^	6.7 (4.9) ^c***^	7.6 (5.5) ^c**^	10.6 (6.4)	3	22.7	*p* <.001
Emotional neglect	*M* (*SD*)	4.3 (4.9) ^a**, b***, c***^	5.8 (5.2) ^b***, c***^	8 (5.7)	9.3 (5.5)	3	34.2	*p* <.001
Physical neglect	*M* (*SD*)	4.9 (5.1) ^a*, b***, c***^	6.4 (5.7) ^c***^	8.4 (5.6)	9.9 (5.4)	3	29.2	*p* <.001
**Depression**								
Early Life Trauma	*M* (*SD*)	4.1 (4.2) ^a***, b***, c***^	6.2 (5.4) ^c***^	6.7 (5.3) ^c***^	10.5 (6.1)	3	64.6	*p* <.001
Emotional abuse	*M* (*SD*)	4.5 (4.5) ^a***, b***, c***^	7.6 (5.8) ^c***^	8.1 (5.5) ^c***^	11.6 (5.6)	3	72.5	*p* <.001
Physical abuse	*M* (*SD*)	5.7 (7.2) ^b***, c***^	7.2 (5.5) ^b*, c***^	9.9 (6.0) ^c**^	13.2 (5.2)	3	46.5	*p* <.001
Sexual abuse	*M* (*SD*)	5.9 (5.4) ^b***, c***^	7.1 (5.2) ^c***^	9.1 (6.1)	10.9 (6.3)	3	19.9	*p* <.001
Emotional neglect	*M* (*SD*)	4.5 (4.6) ^a***, b***, c***^	6.6 (5.3) ^b***, c***^	9.6 (5.9)	11.1 (6.1)	3	64.3	*p* <.001
Physical neglect	*M* (*SD*)	5.5 (5.2) ^a**, b***, c***^	7.4 (5.7) ^c***^	9.2 (6.1)	11.3 (5.8)	3	35.1	*p* <.001
**Stress**								
Early Life Trauma	*M* (*SD*)	6.4 (4.7) ^a***, b***, c***^	8.4 (5.1) ^c***^	8.7 (5.1) ^c***^	11.8 (5.1)	3	49.7	*p* <.001
Emotional abuse	*M* (*SD*)	6.6 (4.7) ^a***, b***, c***^	9.6 (5.1) ^c***^	9.9 (4.9) ^c***^	13.1 (4.6)	3	67.9	*p* <.001
Physical abuse	*M* (*SD*)	7.9 (5.1) ^b***, c***^	9.3 (5.1) ^c**^	10.8 (5.5) ^c*^	13.2 (4.5)	3	24.8	*p* <.001
Sexual abuse	*M* (*SD*)	7.9 (5.2) ^a**, b**, c***^	9.8 (4.8) ^c*^	10.2 (5.1)	12.1 (5.5)	3	16.4	*p* <.001
Emotional neglect	*M* (*SD*)	7.0 (5.0) ^a***, b***, c***^	8.7 (5.0) ^b***, c***^	10.9 (5.2)	11.7 (5.1)	3	36.8	*p* <.001
Physical neglect	*M* (*SD*)	7.8 (5.1) ^a*, b***, c***^	9.3 (5.5) ^c**^	10.5 (5.0)	11.7 (5.3)	3	18.6	*p* <.001
**Negative Emotional states**								
Early Life Trauma	*M* (*SD*)	14.1 (12) ^a***, b***, c***^	20.1 (14.4) ^c***^	21.1 (14.1) ^c***^	31.4 (15.3)	3	65.4	*p* <.001
Emotional abuse	*M* (*SD*)	15.1 (12.5) ^a***, b***, c***^	23.8 (15.2) ^c***^	25.4 (14.4) ^c***^	34.9 (13.9)	3	80.1	*p* <.001
Physical abuse	*M* (*SD*)	18.7 (14.1) ^b***, c***^	23.2 (14.9) ^c***^	28.6 (16.3) ^c**^	37.9 (13.5)	3	40.4	*p* <.001
Sexual abuse	*M* (*SD*)	18.9 (14.5) ^a*, b***, c***^	23.6 (13.6) ^c***^	26.9 (15.7)	33.6 (17.0)	3	23	*p* <.001
Emotional neglect	*M* (*SD*)	15.7 (13.3) ^a***, b***, c***^	21.1 (14.1) ^b***, c***^	28.4 (15.2)	32.1 (15.2)	3	53	*p* <.001
Physical neglect	*M* (*SD*)	18.3 (14.1) ^a**, b***, c***^	23 (15.5) ^c***^	28.1 (15.2)	32.9 (15.6)	3	32.4	*p* <.001
**Self-efficacy**								
Early Life Trauma	*M* (*SD*)	30.3 (5.1) ^a**, b**, c***^	28.7 (4.5) ^c***^	28.6 (4.7) ^c**^	26.5 (6.2)	3	22.4	*p* <.001
Emotional abuse	*M* (*SD*)	29.9 (4.9) ^a**, b***, c***^	28.3 (4.8) ^c***^	27.4 (5.5)	25.8 (6.2)	3	24.4	*p* <.001
Physical abuse	*M* (*SD*)	29.3 (4.9) ^b***, c***^	28.5 (5.2) ^c***^	26.7 (6.0) ^c**^	23.4 (6.2)	3	28	*p* <.001
Sexual abuse	*M* (*SD*)	29.17(5) ^b***, c***^	28.7 (5.1) ^b*, c*^	26.5 (5.6)	25.9 (7.3)	3	11.6	*p* <.001
Emotional neglect	*M* (*SD*)	30.1(5.1) ^a***, b***, c***^	28.4 (4.5) ^b*, c**^	26.8 (5.1)	26.1 (6.7)	3	26.2	*p* <.001
Physical neglect	*M* (*SD*)	29.4(4.9) ^b**, c***^	28.2 (5.1) ^c***^	27.2 (6.4) ^c*^	24.8 (6.0)	3	22.3	*p* <.001
**Anxious Attachment**								
Early Life Trauma	*M* (*SD*)	26.2 (15) ^a***, b***, c***^	34.3 (17.3)	37.1 (14.6)	37.7 (14.6)	3	30.5	*p* <.001
Emotional abuse	*M* (*SD*)	28.6 (16) ^a***, b***, c***^	35.9 (15.7) ^c*^	37.2 (13.2)	40.9 (15.2)	3	28.6	*p* <.001
Physical abuse	*M* (*SD*)	31.4 (16.4) ^a*, b***, c*^	38.6 (16.7)	39.6 (14.7)	38.3 (11.9)	3	10.5	*p* <.001
Sexual abuse	*M* (*SD*)	31.5 (16.5) ^b***^	35.5 (15.1)	39.9 (14.7)	35.2 (14.2)	3	7.9	*p* <.001
Emotional neglect	*M* (*SD*)	27.5 (15.5) ^a***, b***, c***^	36.4 (16.2)	38.4 (14.8)	38.7 (14.3)	3	32	*p* <.001
Physical neglect	*M* (*SD*)	30.6 (16.5) ^a***, b**, c**^	38.6 (15.5)	38.2 (14.2)	37.8 (12.1)	3	14.8	*p* <.001
**Avoidant Attachment**								
Early Life Trauma	*M* (*SD*)	55.2 (19.0) ^a***, b***, c***^	70.8 (20.2) ^b***, c***^	79.4 (18.2) ^c***^	92.2 (19.2)	3	164.2	*p* <.001
Emotional abuse	*M* (*SD*)	61.9 (21.5) ^a***, b***, c***^	77.1(20.9) ^b*, c***^	84.9 (18.2)	91.9 (20.4)	3	91.9	*p* <.001
Physical abuse	*M* (*SD*)	67.1 (22.2) ^a***, b***, c***^	83.9 (18) ^c**^	92.4 (18.7)	100.6 (14.4)	3	74.7	*p* <.001
Sexual abuse	*M* (*SD*)	68.3 (23.2) ^a**, b***, c***^	76.8 (23.2) ^b*, c**^	86.8 (21.5)	90.3 (17.1)	3	30.3	*p* <.001
Emotional neglect	*M* (*SD*)	57.0 (19.3) ^a***, b***, c***^	77.6 (18.0) ^b***, c***^	90.6 (19)	95.6 (16.1)	3	200.8	*p* <.001
Physical neglect	*M* (*SD*)	64.9 (21.7) ^a***, b***, c***^	81.6 (19.9) ^c***^	87.9 (17.8) ^c***^	101.6 (13.9)	3	103.6	*p* <.001

Different letters point out significant differences between groups (***p <.001; **p <.01; *p <.05), (a = Slight to moderate; b = moderate to severe; c = Severe to extreme)

### Relationship between all Variables of the Study

**Fig. 2:**
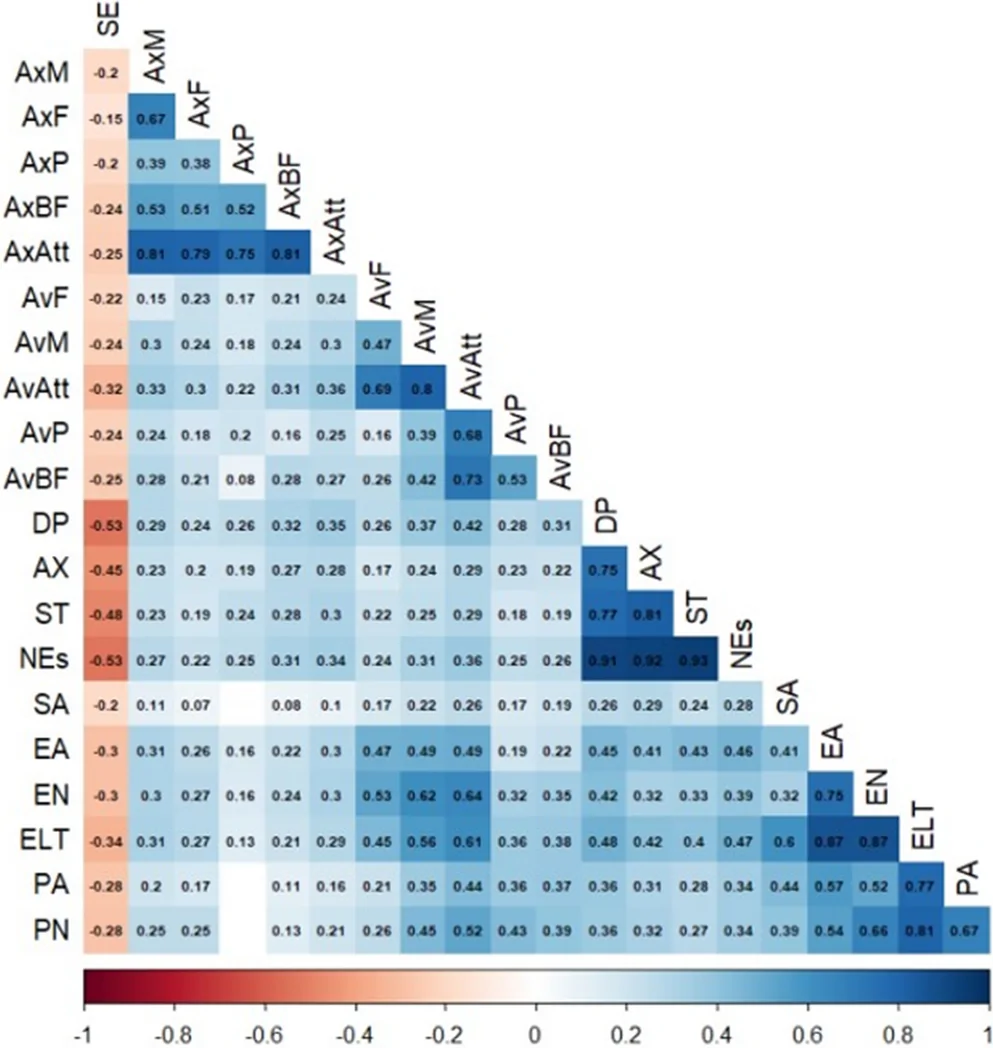
Correlations between Early Life Trauma, Negative Emotional states, Self-efficacy and Attachment styles. **Note.** AX, Anxiety; AvBF, Avoidant attachment Best Friend; AxBF, Anxious attachment Best Friend; AvF, Avoidant attachment Father; AxF, Anxious attachment Father; AvM, Avoidant attachment Mother; AxM, Anxious attachment Mother; AvP, Avoidant attachment Partner; AxP, Anxious attachment Partner; DP, Depression; ELT, Early Life Trauma; EA, Emotional Abuse; EN, Emotional Neglect; NEs, Negative Emotional states; PA, Physical Abuse; PN, Physical Neglect; SE, Self-efficacy; ST, Stress

Fig. 2 shows the significant relationships found between most of the variables (*p* <.05). Only the relationships between anxious attachment with a partner and sexual and physical abuse and physical neglect were not significant (*p* >.05).

### Second-order Confirmatory Factor Analysis

A second-order confirmatory factor analysis (CFA) was performed to assess whether all factors aligned with the overarching construct of ELT. In this model, each item loaded onto the first-order factors, which in turn loaded onto a second-order factor representing the general concept of ELT.

**Fig. 3:**
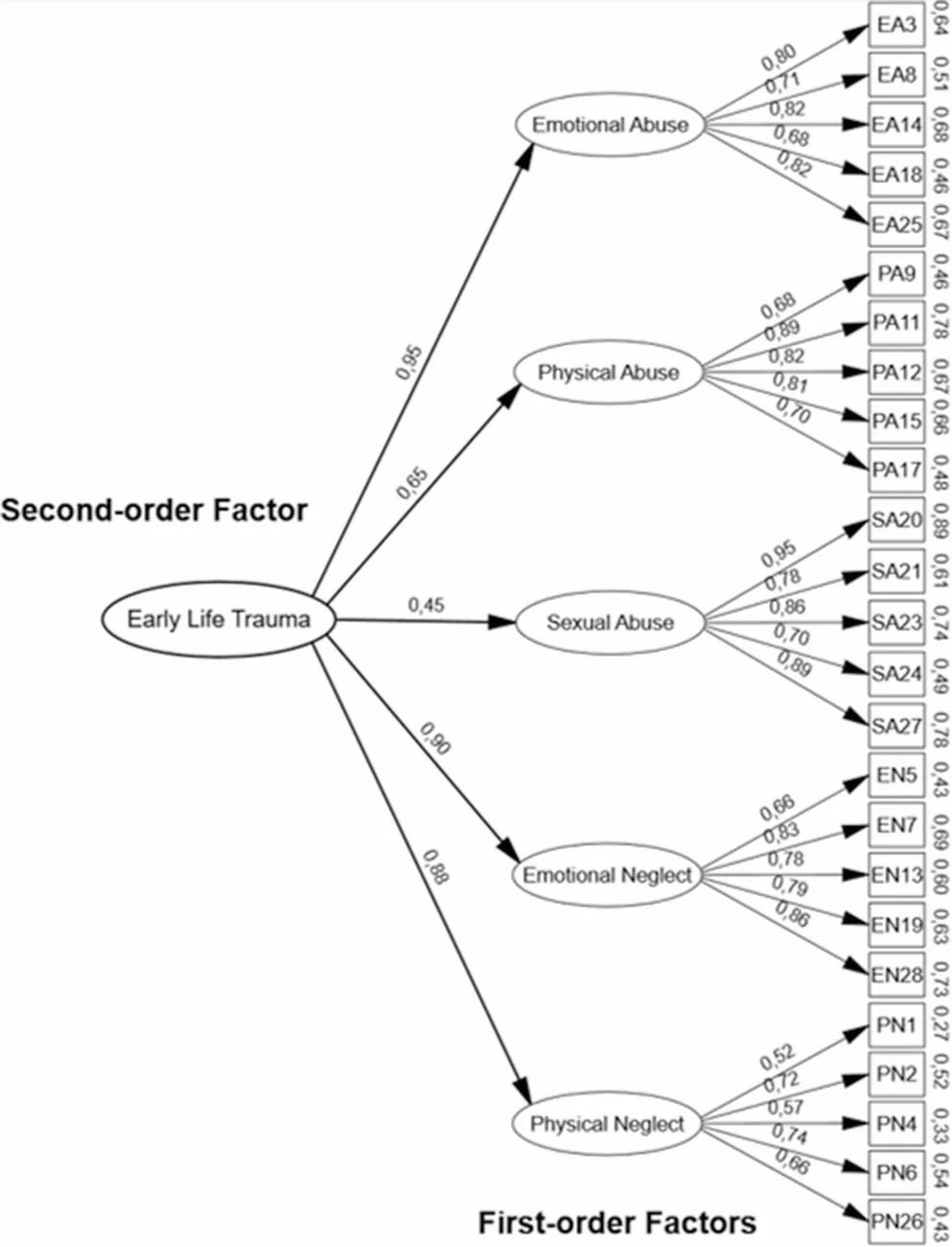
Second-order Confirmatory Factor Analysis of Childhood Trauma Questionnaire

To enhance the model’s fit, modification indices were examined. These indices recommended adding covariances between observed variables within the same factor, specifically items 8–14 of the emotional abuse, 9–17 of the physical abuse, 21–27 of the sexual abuse, 13–19 of the emotional neglect, and items 2–6 of the physical neglect subscale. The second-order CFA results suggested an acceptable fit (CMIN/DF = 6.45; CFI = 0.91; TLI = 0.90; RMSEA = 0.08, CI = 0.07–0.08.07.08). Moreover, factor loadings are statistically significant (*p* <.001) (Fig. 3).

### Structural Equation Model

**Fig. 4:**
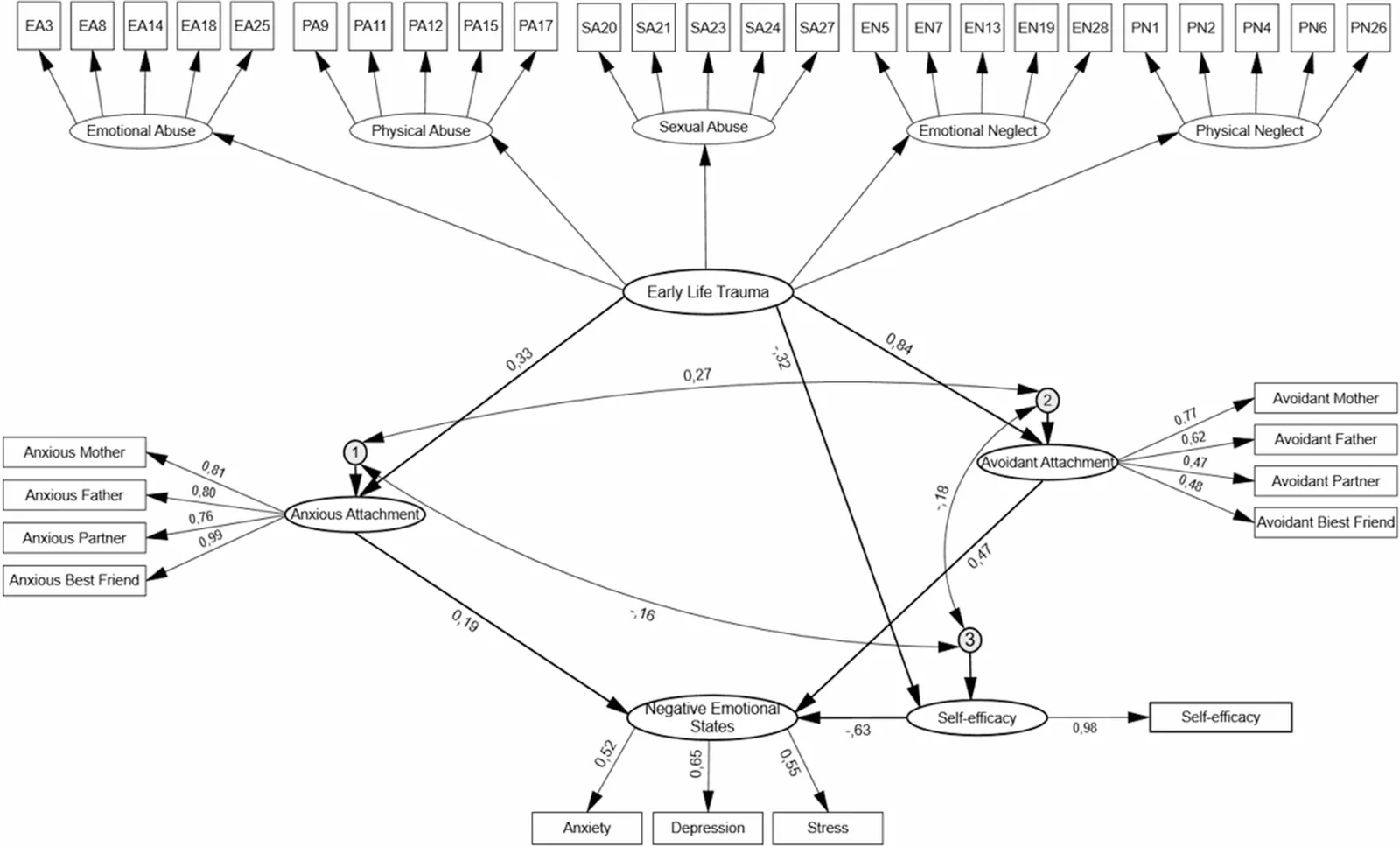
Structural Equation Model of relations between Early Life Trauma, Psychological symptoms, Attachment styles, and Self-efficacy. **Note.**The covariancearrows between observed variables and respective results are not displayed in the figure. The relationship between anxious and avoidant attachment and self-efficacy was significant (*p*<.01)

The results of the Structural Equation Model (Fig. 4) indicated an acceptable fit (CMIN/DF = 4.33; GFI = 0.86; CFI = 0.92; TLI = 0.91; RMSEA = 0.06). Additionally, findings indicated that ELT has a significant impact on the mediating variables, and the mediating variables were significantly related with the development of psychological symptoms (*p* <.01).

### Mediation Analysis

Table 3 shows that the total effect of ELT on psychological symptoms was significant (*ß* = 0.49; 95% CI [0.43–0.55.43.55]; *t* = 16.28; *p* <.001; *R*^*2*^ = 22%). Similarly, the direct effects of ELT, including emotional abuse, physical abuse, sexual abuse, emotional neglect, and physical neglect, on general psychological symptoms in adulthood were also significant (*p* <.01). Concerning the bootstrap analysis, the results revealed that the indirect effect through self-efficacy was statistically significant (*ß* = 0.34; 95% CI [0.28–0.40.28.40]; *t* = 11.85; *p* <.001; *R*^*2*^ = 38%), indicating that self-efficacy acted as a partial mediator.

**Table 3:** Unstandardized and Standardized effects from the Mediation Model

Independent Variables		Mediators		Dependent Variables	Direct Effect						Indirect Effect *ß*	95% CI	
					Unstandardized *ß* (SE)			Standardized *ß*					
Early Life Trauma	➝	Self-efficacy	➝	Negative Emotional states	− 0.13 (0.01)***	-1.19 (0.08)***	0.34 (0.03)***	− 0.34	− 0.42	0.33	0.15	0.11	0.19
Emotional Abuse	➝				− 0.35 (0.04)***	-1.23 (0.08)***	1.12 (0.09)***	− 0.30	− 0.43	0.34	0.42	0.31	0.55
Physical Abuse	➝				− 0.51 (0.06)***	-1.34 (0.08)***	1.11 (0.15)***	− 0.28	− 0.47	0.21	0.69	0.48	0.90
Sexual Abuse	➝				− 0.36 (0.06)***	-1.41 (0.08)***	0.95 (0.14)***	− 0.20	− 0.49	0.19	0.50	0.31	0.70
Emotional Neglect	➝				− 0.33 (0.04)***	-1.30 (0.08)***	− 0.82 (0.09)***	− 0.30	− 0.45	0.26	0.43	0.32	0.56
Physical Neglect	➝				− 0.48 (0.05)***	-1.34 (0.08)***	1.02 (0.14)***	− 0.28	− 0.47	0.21	0.65	0.49	0.82
Early Life Trauma	➝	Anxious Attachment	➝	Negative Emotional states	0.32 (0.03)***	0.21 (0.03)***	0.42 (0.03)***	0.29	0.22	0.41	0.07	0.04	0.09
Emotional Abuse	➝				1.06 (0.11)***	0.20 (0.03)***	1.33 (0.10)***	0.30	0.22	0.40	0.22	0.14	0.30
Physical Abuse	➝				0.92 (0.18)***	0.27 (0.03)***	1.55 (0.16)***	0.16	0.29	0.30	0.25	0.16	0.36
Sexual Abuse	➝				0.54 (0.18)**	0.29 (0.03)***	1.29 (0.15)***	0.10	0.31	0.25	0.16	0.07	0.27
Emotional Neglect	➝				1.04 (0.11)***	0.23 (0.03)***	1.02 (0.10)***	0.30	0.24	0.32	0.24	0.16	0.32
Physical Neglect	➝				1.08 (0.17)***	0.26 (0.03)***	1.39 (0.15)***	0.21	0.28	0.29	0.28	0.19	0.39
Early Life Trauma	➝	Avoidant Attachment	➝	Negative Emotional states	0.99 (0.04)***	0.08 (0.02)***	0.41 (0.04)***	0.61	0.12	0.40	0.08	0.03	0.13
Emotional Abuse	➝				2.56 (0.15)***	0.11 (0.02)***	1.26 (0.11)***	0.49	0.18	0.38	0.29	0.18	0.41
Physical Abuse	➝				3.59 (0.24)***	0.17 (0.02)***	1.2 (0.17)***	0.44	0.26	0.23	0.60	0.44	0.77
Sexual Abuse	➝				2.05 (0.25)***	0.20 (0.02)***	1.04 (0.16)***	0.26	0.31	0.20	0.41	0.30	0.54
Emotional Neglect	➝				3.23 (0.13)***	0.12 (0.02)***	0.86 (0.12)***	0.64	0.19	0.27	0.40	0.23	0.56
Physical Neglect	➝				3.95 (0.21)***	0.16 (0.02)***	1.04 (0.17)***	0.52	0.25	0.21	0.64	0.47	0.82

***p < .001; **p < .01; *p < .05; results sequence: ELT - > Mediator; Mediator -> Psychological Symptoms; ELT -> Negative Emotional states

Moreover, the analysis demonstrated a significant total effect of emotional abuse on psychological symptoms (*ß* = 1.55; 95% CI [1.36–1.74]; *t* = 16.02; *p* <.001; *R*^*2*^ = 22%). The bootstrap analysis indicated that the effects via self-efficacy (*ß* = 1.12; 95% CI [0.95 − 1.3]; *t* = 12.5; *p* <.001; *R*^*2*^ = 39%), anxious attachment (*ß* = 0.20; 95% CI [0.15–0.26.15.26]; *t* = 7.39; *p* <.001; *R*^*2*^ = 26%), and avoidant attachment (*ß* = 0.11; 95% CI [0.07–0.15.07.15]; *t* = 5.43; *p* <.001; *R*^*2*^ = 24%) were significant, suggesting that these factors served as partial mediators.

For physical abuse, there was also a significant total effect on psychological symptoms (*ß* = 1.8; 95% CI [1.49–2.12]; *t* = 11.2; *p* <.001; *R*^*2*^ = 12%). The bootstrap analysis revealed significant indirect effects via self-efficacy (*ß* = 1.11; 95% CI [0.83 − 1.4]; *t* = 7.59; *p* <.001; *R*^*2*^ = 32%), anxious attachment (*ß* = 1.55; 95% CI [1.25–1.86]; *t* = 10.00; *p* <.001; *R*^*2*^ = 20%), and avoidant attachment were statistically significant (*ß* = 1.2; 95% CI [0.86–1.54.86.54]; *t* = 6.91; *p* <.001; *R*^*2*^ = 17%), indicating partial mediators.

In the case of sexual abuse, the total effects on psychological symptoms were significant (*ß* = 1.45; 95% CI [1.14–1.77]; *t* = 9.05; *p* <.001; *R*^*2*^ = 8%). Bootstrap analysis showed that the indirect effect via self-efficacy (*ß* = 0.95; 95% CI [0.67–1.23.67.23]; *t* = 6.74; *p* <.001; *R*^*2*^ = 31%) and avoidant attachment (*ß* = 1.04; 95% CI [0.73–1.35.73.35]; *t* = 10.08; *p* <.001; *R*^*2*^ = 17%) were significant, identifying both as partial mediators.

For emotional neglect, the total effect on psychological symptoms was also significant (*ß* = 1.25; 95% CI [1.06–1.44]; *t* = 12.92; *p* <.001; *R*^*2*^ = 15%). Bootstrap analysis revealed significant indirect effect via self-efficacy (*ß* = 0.82; 95% CI [0.65–0.99.65.99]; *t* = 9.17; *p* <.001; *R*^*2*^ = 34%), anxious attachment (*ß* = 1.01; 95% CI [0.82–1.21.82.21]; *t* = 10.32; *p* <.001; *R*^*2*^ = 20%), and avoidant attachment were statistically significant (*ß* = 0.86; 95% CI [0.61 − 1.10]; *t* = 6.85; *p* <.001; *R*^*2*^ = 17%), indicating partial mediators.

Finally, for physical neglect, the effect on psychological symptoms was significant (*ß* = 1.67; 95% CI [1.38–1.97]; *t* = 11.23; *p* <.001; *R*^*2*^ = 12%). Bootstrap analysis indicated significant indirect effects via self-efficacy (*ß* = 0.25; 95% CI [0.15–0.35.15.35]; *t* = 4.99; *p* <.001; *R*^*2*^ = 25%), anxious attachment (*ß* = 1.39; 95% CI [1.11–1.68]; *t* = 9.54; *p* <.001; *R*^*2*^ = 19%), and avoidant attachment (*ß* = 1.04; 95% CI [0.70–1.37.70.37]; *t* = 6.10; *p* <.001; *R*^*2*^ = 17%), confirming these as partial mediators (for details, see Supplementary Information Table SII).

## Discussion

This study sought to investigate the mediating effects of self-efficacy and attachment styles on the connection between ELT and NEs among a substantial cohort of the general population in Portugal.

Consistent with previous studies, our results showed that individuals who experienced ELT exhibited higher levels of NEs, as well as lower scores of self-efficacy, and a higher prevalence of anxious and avoidant attachment styles. These results align with previous studies that have shown a link between childhood neglect and abuse and increased psychological vulnerability, mediated by diminished self-efficacy (Betz et al., 2021; Sachs-Ericsson et al., 2011). Additionally, our results support previous findings that ELT is associated with insecure attachment styles, as early neglect and abuse often contribute to maladaptive attachment patterns in adulthood (Erozkan, 2016). Furthermore, Karna and Simon (2024) already showed a relationship between negative emotion regulation strategies, anxious and avoidant attachment styles, and ELT experiences.

The current study also provided partial support for the hypothesis that self-efficacy and attachment styles mediate the relationship between ELT and NEs. These findings highlight the broad implications of ELT on multiple dimensions of psychological functioning. Importantly, individuals with lower self-efficacy tended to report more maladaptive attachment styles, a relationship corroborated by previous studies. Deniz and Kurtuluş (2023) found that higher self-efficacy was associated with secure attachment styles and less anxious-avoidant patterns, reinforcing the mediating role of self-efficacy between attachment and life satisfaction. Similarly, Morison and Benight (2022) identified self-efficacy as a key mediator between attachment styles and the development of post-traumatic stress disorder symptoms.

Our results also demonstrated a strong relationship between self-efficacy and the development of NEs, such as anxiety, depression, and stress. This aligns with the literature, which suggests that individuals with lower self-efficacy are more likely to experience psychological symptoms (Huang et al., 2023). Türk-Kurtça and Kocatürk (2020) similarly found that emotional self-efficacy, internal locus of control, and psychological resilience are influenced by childhood trauma and play a role in mitigating psychological distress. Guerra et al. (2018) also reported a negative association between self-efficacy and psychological symptoms, further emphasizing the importance of family support in fostering self-efficacy and reducing psychological distress.

In addition to self-efficacy, maladaptive attachment styles with significant figures (e.g., mother, father, partner, best friend) were found to negatively impact NEs in adulthood. These findings are consistent with studies showing that insecure attachment styles in adulthood (anxious and avoidant) are positively associated with psychological distress and negatively related to secure childhood attachment (Shen et al., 2021). Trucharte et al. (2022) also demonstrated that anxious and avoidant attachment styles are related to psychological well-being and mental health symptoms, with avoidant attachment having a direct effect on symptoms and anxious attachment having an indirect effect through psychological mediating processes.

The literature consistently identifies physical and emotional abuse and neglect in childhood as significant risk factors for mental health problems in adulthood. Our findings suggest that interventions for individuals with psychological symptoms who have experienced ELT may benefit from focusing on enhancing self-efficacy and addressing insecure attachment styles. However, further research is needed to explore these relationships more comprehensively, particularly in terms of different dimensions of self-efficacy and attachment styles. While the current study reveals significant associations between these variables, the effects are relatively weak, especially in the relationship between ELT, self-efficacy, and anxious attachment.

Understanding the complex interplay between self-efficacy, attachment styles, and ELT requires considering both direct and indirect relationships among these variables. The discrepancy between the univariate correlations and the SEM findings underscores the significance of accounting for shared variation and indirect effects. Univariate studies revealed a direct correlation between self-efficacy and attachment patterns, whereas SEM considers the simultaneous impact of many factors. The incorporation of ELT as a principal predictor may have attenuated the direct relationship between self-efficacy and attachment patterns, as ELT exerts an independent influence on both variables. Moreover, self-efficacy and attachment styles may operate as independent mediators between ELT and negative emotional states, rather than being directly associated. Considering these complexity, future study should investigate potential indirect pathways or moderating effects to more accurately elucidate the nuanced interactions among these variables.

### Implications and Future Directions

The results of this study hold significant significance for clinical practice and therapy. Strategies designed to enhance self-efficacy should be prioritized in trauma-informed therapies for individuals with a history of early childhood trauma, since this appears to be a vital factor in mitigating the adverse psychological effects of early trauma. Modalities like cognitive-behavioral therapy, which emphasize improving self-efficacy and promoting adaptive coping strategies, may be particularly effective. Moreover, interventions aimed at improving attachment security, such as attachment-based therapy, can aid individuals in reinterpreting their interpersonal relationships and alleviating feelings of anxiety, depression, and stress.

The present study has yielded significant insights into the mediating roles of self-efficacy and attachment types; however, additional research is required to examine these interactions more comprehensively. Future research should examine the various components of self-efficacy (e.g., emotional, cognitive, and social) and their distinct roles in the development of NEs. Furthermore, longitudinal research may elucidate the causal relationships among ELT, self-efficacy, attachment styles, and NEs across time. Future research should also use other type of measures, in order to include other types of attachment styles, such as the disorganized.

### Limitations

Despite these significant results, several limitations must be acknowledged. The use of self-report surveys may introduce bias, as individuals’ recollections of traumatic situations are subjective and susceptible to memory mistakes or social desirability effects. Although the instruments exhibited strong internal consistency, the dependence on self-reports may restrict the generalizability of the results. Another limitation is the sample’s homogeneity, especially regarding gender, marital status, and education level, which may influence the generalizability of the findings across various populations. Another limitation of this study is the employment of snowball sampling, a non-probability sampling method, which may restrict the sample’s representativeness. Consequently, prudence must be observed when extrapolating the results to the wider community. Although inferential statistical studies were performed to examine correlations among variables, the results must be interpreted with the awareness that the sample was not randomly selected. To strengthen the validity of future research, integrating objective measures, like clinical interviews or physiological evaluations and probability sampling approaches could yield a more thorough comprehension of the effects of ELT on psychological functioning.

## Conclusion

In conclusion, this study adds to the growing body of evidence demonstrating the significant roles of self-efficacy and attachment styles in the relationship between early life trauma and negative emotional states. By highlighting the mediating effects of these factors, the findings underscore the importance of targeting self-efficacy and attachment in trauma-informed interventions. Although the relationships between ELT, self-efficacy, and attachment styles were statistically significant, the effect sizes were relatively modest, indicating that more research is needed to fully understand these complex dynamics. Future studies should consider diverse methodologies and explore the longitudinal effects of ELT on psychological outcomes to inform more effective therapeutic interventions for individuals affected by early trauma.

## Supplementary Information

Below is the link to the electronic supplementary material.

Supplementary Material 1 (DOCX 76.7 KB)

## Data Availability

The data that support the findings of this study are available from the corresponding author, upon reasonable request.

## References

[CR1] Aluh, D. O., Azeredo-Lopes, S., Cardoso, G., Pedrosa, B., Grigaitė, U., Dias, M., Xavier, M., & Caldas-de-Almeida, J. M. (2022). Social anxiety disorder and childhood adversities in Portugal: Findings from the WHO world mental health survey initiative. *Psychiatry Research,**315*(114734), 1–9. 10.1016/j.psychres.2022.114734 35872402

[CR2] Aguirre-Urreta, M. I., & Hu, J. (2019). Detecting common method bias: Performance of the Harman’s single-factor test. *Database for Advances in Information Systems, 50*(2), 45–70.

[CR3] Alfons, A., Ateş, N. Y., & Groenen, P. J. F. (2021). A robust bootstrap test for mediation analysis. *Organizational Research Methods, 25*(3), 591–617. 10.1177/1094428121999096

[CR4] Anderson, J. C., & Gerbing, D. W. (1988). Structural equation modeling in practice: A review and recommended two-step approach. *Psychological Bulletin,**103*(3), 411–423. 10.1037/0033-2909.103.3.411

[CR5] Bandura, A. (1997). *Self-Efficacy: The Exercise of Control*. W H Freeman & Company.

[CR6] Bernstein, D. P., Stein, J. A., Newcomb, M. D., Walker, E., Pogge, D., Ahluvalia, T., Stokes, J., Handelsman, L., Medrano, M., Desmond, D., & Zule, W. (2003). Development and validation of a brief screening version of the childhood trauma questionnaire. *Child Abuse & Neglect,**27*(2), 169–190. 10.1016/S0145-2134(02)00541-0 12615092

[CR7] Betz LT, Penzel N, Rosen M, Kambeitz J. (2021). Relationships between childhood trauma and perceived stress in the general population: a network perspective.* Psychological Medicine, 51*(15), 2696–2706. 10.1017/S003329172000135X 32404227

[CR8] Bockting, W., Belloir, J., Jackman, K., & Fabiano, F. (2024). General self‐efficacy as a mediator of the association between adverse childhood experiences and psychological distress in gender‐minority individuals. *Journal of Nursing Scholarship, 56*(1), 9–17. 10.1111/jnu.12889 36935475

[CR9] Bosmans, G., Braet, C., & Van Vlierberghe, L. (2010). Attachment and symptoms of psychopathology: Early maladaptive schemas as a cognitive link? *Clinical Psychology & Psychotherapy,**17*(5), 374–385. 10.1002/cpp.667 20013761

[CR10] Brown, T. A. (2006). Confirmatory factor analysis for applied research (1st ed.). Guilford.

[CR11] Byrne, B. M. (2011). *Structural equation modeling with Mplus: Basic concepts, applications, and programming* (1st ed). Routledge.

[CR12] Cantazaro, A., & Wei, M. (2010). Adult attachment, dependence, self-criticism, and depressive symptoms: A test of a mediational model. *Journal of Personality*, *78*(4), 1135–1162. 10.1111/j.1467-6494.2010.00645.x 20545820

[CR13] Clear, S. J., Gardner, A. A., Webb, H. J., & Zimmer-Gembeck, M. J. (2020). Common and distinct correlates of depression, anxiety, and aggression: Attachment and emotion regulation of sadness and anger. *Journal of Adult Development,**27*(3), 181–191. 10.1007/s10804-019-09333-0

[CR14] Cohrdes, C., & Mauz, E. (2020). Self-Efficacy and emotional stability buffer negative effects of adverse childhood experiences on young adult Health-Related quality of life. *Journal of Adolescent Health*, *67*(1), 93–100. 10.1016/j.jadohealth.2020.01.005 32192828

[CR15] Corcoran, M., & McNulty, M. (2018). Examining the role of attachment in the relationship between childhood adversity, psychological distress and subjective well-being. *Child Abuse & Neglect,**76*, 297–309. 10.1016/j.chiabu.2017.11.012 29175733

[CR16] Degnan, A., Berry, K., Humphrey, C., & Bucci, S. (2022). The role of attachment and dissociation in the relationship between childhood interpersonal trauma and negative symptoms in psychosis. *Clinical Psychology and Psychotherapy*, *29*(5), 1692–1706. 10.1002/cpp.2731 35218114 PMC9790513

[CR17] Dewi, Y., & Indra, I. (2024). Journal of applied management research navigating consumer behaviour: Exploring the influence of fashion trends and social media on gen Z and millennial consumption in West Java. *Journal of Applied Management Research*, *4*(1), 1–9. 10.36441/jamr

[CR18] Donisi, V., Perlini, C., Mazzi, M. A., Rimondini, M., Garbin, D., Ardenghi, S., Rampoldi, G., Montelisciani, L., Antolini, L., Strepparava, M. G., & Del Piccolo, L. (2022). Training in communication and emotion handling skills for students attending medical school: Relationship with empathy, emotional intelligence, and attachment style. *Patient Education and Counseling*, *105*(9), 2871–2879. 10.1016/j.pec.2022.05.015 35715300

[CR19] Erozkan, A. (2016). The link between types of attachment and childhood trauma. *Universal Journal of Educational Research, 4*(5), 1071–1079. 10.13189/ujer.2016.040517

[CR20] Fornell, C., & Larcker, D. F. (1981). Structural equation models with unobservable variables and measurement error: Algebra and statistics. *Journal of Marketing Research*, *18*(3), 382–388. 10.2307/3150980

[CR21] Fuchshuber, J., Hiebler-Ragger, M., Kresse, A., Kapfhammer, H. P., & Unterrainer, H. F. (2019). The influence of attachment styles and personality organization on emotional functioning after childhood trauma. *Frontiers in Psychiatry*, *10*(643), 1–10. 10.3389/fpsyt.2019.00643 31543844 PMC6739441

[CR22] Glaesmer, H. (2016). Assessing childhood maltreatment on the population level in Germany: Findings and methodological challenges. *Child and Adolescent Psychiatry and Mental Health,**10*(15), 1–6. 10.1186/s13034-016-0104-9 27303440 PMC4906889

[CR23] Gormley, B., & McNiel, D. E. (2010). Adult attachment orientations, depressive symptoms, anger, and self-directed aggression by psychiatric patients. *Cognitive Therapy and Research*, *34*(3), 272–281. 10.1007/s10608-009-9267-5

[CR24] Haj-Yahia, M. M., Sokar, S., Hassan-Abbas, N., & Malka, M. (2019). The relationship between exposure to family violence in childhood and post-traumatic stress symptoms in young adulthood: The mediating role of social support. *Child Abuse and Neglect*, *92*, 126–138. 10.1016/j.chiabu.2019.03.023 30974256

[CR25] Henry, J. D., & Crawford, J. R. (2005). The short-form version of the depression anxiety stress scales (DASS-21): Construct validity and normative data in a large non-clinical sample. *British Journal of Clinical Psychology*, *44*(2), 227–239. 10.1348/014466505X29657 16004657

[CR26] Henseler, J., Ringle, C. M., & Sarstedt, M. (2014). A new criterion for assessing discriminant validity in variance-based structural equation modeling. *Journal of the Academy of Marketing Science*, *43*(1), 115–135. 10.1007/s11747-014-0403-8

[CR27] Huang, Q., Wang, X., Ge, Y., & Cai, D. (2023). Relationship between self-efficacy, social rhythm, and mental health among college students: A 3-year longitudinal study.* Current psychology, 42*(11), 9053–9062. 10.1007/s12144-021-02160-1 PMC8364412 34413621

[CR28] Karna, A., & Simon, S. (2024). Exploring emotion regulation mechanisms and attachment styles in individuals with childhood traum. *World Journal of Advanced Research and Reviews,**22*(1), 302–311. 10.30574/wjarr.2024.22.1.1061

[CR29] Kessler, R. C., McLaughlin, K. A., Green, J. G., Gruber, M. J., Sampson, N. A., Zaslavsky, A. M., ... & Williams, D. R. (2010). Childhood adversities and adult psychopathology in the WHO World Mental Health Surveys. *The British journal of psychiatry, 197*(5), 378–385. 10.1192/bjp.bp.110.080499 PMC2966503 21037215

[CR30] Kline, R. B. (2016). *Principles and Practice of Structural Equation Modeling* (4th ed). The Guilford Press.

[CR31] Kobak, R. R., Cole, H. E., Ferenz-Gillies, R., Fleming, W. S., & Gamble, W. (1993). Attachment and emotion regulation during mother-teen problem solving: A control theory analysis. *Child Development,**64*(1), 231–245. 10.1111/j.1467-8624.1993.tb02906.x 8436031

[CR32] Komarraju, M., Swanson, J., & Nadler, D. (2014). Increased career Self-Efficacy predicts college students’ motivation, and course and major satisfaction. *Journal of Career Assessment,**22*(3), 420–432. 10.1177/1069072713498484

[CR33] Liu, C., & Ma, J. L. (2019). Adult attachment style, emotion regulation, and social networking sites addiction. *Frontiers in Psychology*, *10*(2352), 1–7. 10.3389/fpsyg.2019.02352 31749729 PMC6843004

[CR34] Mikulincer, M., Shaver, P. R., & Pereg, D. (2003). Attachment theory and affect regulation: The dynamics, development, and cognitive consequences of attachment related strategies. *Motivation and Emotion,**27*(2), 77–102. 10.1023/A:1024515519160

[CR35] Moreira, H., Martins, T., Gouveia, M. J., & Canavarro, M. C. (2015). Assessing adult attachment across different contexts: Validation of the Portuguese version of the experiences in close relationships-relationship structures questionnaire. *Journal of Personality Assessment*, *97*(1), 22–30. 10.1080/00223891.2014.950377 25175516

[CR36] Muller, R. T., Thornback, K., & Bedi, R. (2012). Attachment as a mediator between childhood maltreatment and adult symptomatology. *Journal of Family Violence*, *27*(3), 243–255. 10.1007/s10896-012-9417-5

[CR37] Pinto, R., De Castro, M. V., Silva, L., Jongenelen, I., Maia, A., & Levendosky, A. A. (2021). The impact of psychopathology associated with childhood trauma on quality of life in Portuguese adolescents: A two-wave longitudinal study. *Frontiers in Psychiatry,**12*(650700), 1–8. 10.3389/fpsyt.2021.650700 PMC8517175 34658939

[CR38] Rossi, R., Jannini, T. B., Ciocca, G., Cipriani, C., Socci, V., Pacitti, F., & Di Lorenzo, G. (2023). Attachment and resilience as mediators or moderators in the relationship between trauma and psychotic-like experiences. *Schizophrenia Research*, *258*, 36–44. 10.1016/j.schres.2023.07.005 37473666

[CR39] Ryan, N. E., Solberg, V. S., & Brown, S. D. (1996). Family dysfunction, parental attachment, and career search Self-Efficacy among community college students. *Journal of Counseling Psychology,**43*(1), 84–89. 10.1037/0022-0167.43.1.84

[CR40] Sachs-Ericsson, N., Medley, A. N., Kendall-Tackett, K., & Taylor, J. (2011). Childhood abuse and current health problems among older adults: The mediating role of self-efficacy. *Psychology of violence, 1*(2), 106–120. 10.1037/a0023139 PMC3171165 21922052

[CR41] Scholz, U., Gutiérrez Doña, B., Sud, S., & Schwarzer, R. (2002). Is General Self-Efficacy a Universal Construct? *European Journal of Psychological Assessment,**18*(3), 242–251. 10.1027/1015-5759.18.3.242

[CR42] Shen, F., Liu, Y., & Brat, M. (2021). Attachment, self-esteem, and psychological distress: A multiple-mediator model. *The Professional Counselor, 11*(2), 129–142. 10.15241/fs.11.2.129

[CR43] Silva, A., Ferreira, S., Silva Pinto, É., Rocha, S. A., & Barbosa-Rocha, N. (2023). The relationship between childhood abuse and adult attachment styles: The mediator role of sensory Over-Responsivity. *Journal of Aggression, Maltreatment & Trauma,**33*(2), 236–254. 10.1080/10926771.2023.2186298

[CR44] Simon, H. L. M., DiPlacido, J., & Conway, J. M. (2019). Attachment styles in college students and depression: The mediating role of self differentiation. *Mental Health and Prevention*, *13*, 135–142. 10.1016/j.mhp.2019.01.011

[CR45] Stensen, K., Jozefiak, T., & Lydersen, S. (2022). Psychometric properties of the caregiver-teacher report form in a sample of Norwegian preschool children. *Nordic Journal of Psychiatry*, *76*(8), 616–622. 10.1080/08039488.2022.2027519 35073500

[CR46] Thombs, B. D., Bernstein, D. P., Lobbestael, J., & Arntz, A. (2009). A validation study of the Dutch childhood trauma questionnaire-short form: Factor structure, reliability, and known-groups validity. *Child Abuse & Neglect,**33*(8), 518–523. 10.1016/j.chiabu.2009.03.001 19758699

[CR47] Villada, C., Hidalgo, V., Almela, M., & Salvador, A. (2018). Assessing performance on an evaluated speaking task: The role of self-efficacy, anxiety, and cardiac autonomic reactivity. *Journal of Psychophysiology*, *32*(2), 64–74. 10.1027/0269-8803/a000185

[CR48] Weindl, D., Knefel, M., Glück, T. M., Tran, U. S., & Lueger-Schuster, B. (2018). Motivational capacities after prolonged interpersonal childhood trauma in institutional settings in a sample of Austrian adult survivors. *Child Abuse & Neglect,**76*, 194–203. 10.1016/j.chiabu.2017.11.001 29128740

[CR49] Ye, Z., Wei, X., Zhang, J., Li, H., & Cao, J. (2024). The impact of adverse childhood experiences on depression: The role of insecure attachment styles and emotion dysregulation strategies. *Current Psychology*, *43*(5), 4016–4026. 10.1007/s12144-023-04613-1 PMC10099002 37359705

[CR50] Zhang, Q., Zhang, Q., Ran, G., & Liang, Y. (2023). Childhood abuse and depression in emerging adults: The mediating role of regulatory emotional self-efficacy and the moderating role of subjective social status. *Journal of Adult Development*. 10.1007/s10804-023-09463-6

