# Supplementary material for: Self-Efficacy and Attachment Styles Mediate the Impact of Early Life Trauma on Negative Emotional States in Adults Living in Portugal
Source: J Child Adolesc Trauma. 2026 Jan 8;19(3):1011–25. doi: 10.1007/s40653-025-00812-z (PMC13546191; doi:10.1007/s40653-025-00812-z)
Supplement: Supplementary file 1 [file 40653_2025_812_MOESM1_ESM.docx]

**Supplementary Information (SI)**

**Table SI**

| Subscales | HTMT | | | | | AVE | Cronbach’s Alpha | McDonald’s Omega |
| --- | --- | --- | --- | --- | --- | --- | --- | --- |
|  | Emotional Abuse | Physical Abuse | Sexual Abuse | Emotional Neglect | Physical Neglect |  |  |  |
| Emotional Abuse | - |  |  |  |  | 0.65 | 0.87 | 0.89 |
| Physical Abuse | 0.61 | - |  |  |  | 0.60 | 0.88 | 0.89 |
| Sexual Abuse | 0.44 | 0.48 | - |  |  | 0.68 | 0.90 | 0.92 |
| Emotional Neglect | 0.85 | 0.56 | 0.34 | - |  | 0.62 | 0.89 | 0.88 |
| Physical Neglect | 0.63 | 0.83 | 0.46 | 0.75 | - | 0.42 | 0.75 | 0.79 |
| **Note**. AVE, Average Variance Extracted; HTMT, Heterotrait-Monotrait Ratio | | | | | | | | |

**Table SII** Unstandardized and Standardized effects from the Mediation Model

| Independent  Variables |  | Mediators |  | Dependent Variables | Direct Effect | | | | | | Indirect  Effect  *ß* | | 95%  CI | | |
| --- | --- | --- | --- | --- | --- | --- | --- | --- | --- | --- | --- | --- | --- | --- | --- |
|  |  |  |  |  | Unstandardized  *ß*(SE) | | | Standardized  *ß* | | |  |  |  |  |  |
| Early Life Trauma |  | Self-efficacy |  | Anxiety | -.13  (.01)*** | -.36  (.03)*** | .11  (.01)*** | -.34 | -.35 | .30 | .05 | .03 | | .06 |  |
|  |  |  |  | Depression |  | -.45 (.03)*** | .13  (.01)*** |  | -.42 | .33 | .06 | .04 | | .07 |  |
|  |  |  |  | Stress |  | -.39 (.03)*** | .10  (.01)*** |  | -.39 | .27 | .05 | .04 | | .06 |  |
| Emotional Abuse |  | Self-efficacy |  | Anxiety | -.35  (.04)*** | -.37  (.03)*** | .36  (.03)*** | -.30 | -.37 | .30 | .13 | .09 | | .17 |  |
|  |  |  |  | Depression |  | -.47  (.03)*** | .40  (.03)*** |  | -.44 | .32 | .16 | .12 | | .21 |  |
|  |  |  |  | Stress |  | -.39  (.03)*** | .37  (.03)*** |  | -.39 | .32 | .13 | .10 | | .17 |  |
| Physical Abuse |  | Self-efficacy |  | Anxiety | -.51  (.06)*** | -.41  (.03)*** | .37  (.06)*** | -.28 | -.40 | .20 | .21 | .14 | | .28 |  |
|  |  |  |  | Depression |  | -.50  (.03)*** | .46  (.05)*** |  | -.47 | .23 | .26 | .18 | | .34 |  |
|  |  |  |  | Stress |  | -.44  (.03)*** | .29  (.05)*** |  | -.44 | .16 | .22 | .15 | | .30 |  |
| Sexual Abuse |  | Self-efficacy |  | Anxiety | -.36  (.06)*** | -.42  (.03)*** | .38  (.05)*** | -.20 | -.41 | .20 | .15 | .09 | | .21 |  |
|  |  |  |  | Depression |  | -.53  (.03)*** | .31  (.05)*** |  | -.50 | .16 | .19 | .11 | | .27 |  |
|  |  |  |  | Stress |  | -.45  (.03)*** | .26  (.05)*** |  | -.45 | .15 | .16 | .10 | | .22 |  |
| Emotional Neglect |  | Self-efficacy |  | Anxiety | -.33  (.04)*** | -.40  (.03)*** | .24  (.03)*** | -.30 | -.39 | .21 | .13 | .10 | | .17 |  |
|  |  |  |  | Depression |  | -.48  (.03)*** | .35  (.03)*** |  | -.45 | .29 | .16 | .12 | | .20 |  |
|  |  |  |  | Stress |  | -.42  (.03)*** | .23  (.03)*** |  | -.42 | .21 | .14 | .10 | | .18 |  |
| Physical Neglect |  | Self-efficacy |  | Anxiety | -.48  (.05)*** | -.40  (.03)*** | .37  (.05)*** | -.28 | -.39 | .21 | .20 | .14 | | .25 |  |
|  |  |  |  | Depression |  | -.50  (.03)*** | .41  (.05)*** |  | -.47 | .22 | .24 | .18 | | .31 |  |
|  |  |  |  | Stress |  | -.44  (.03)*** | .25  (.05)*** |  | -.44 | .15 | .21 | .16 | | .27 |  |
| Early Life Trauma |  | Anxious  Attachment |  | Anxiety | .32  (.03)*** | .06  (.01)*** | .14  (.01)*** | .29 | .18 | .37 | .02 | .01 | | .03 |  |
|  |  |  |  | Depression |  | .08  (.01)*** | .16  (.01)*** |  | .23 | .41 | .03 | .02 | | .04 |  |
|  |  |  |  | Stress |  | .07  (.01)*** | .13  (.01)*** |  | .20 | .34 | .02 | .01 | | .03 |  |
| Emotional Abuse |  | Anxious  Attachment |  | Anxiety | 1.06  (.11)*** | .06  (.01)*** | .42  (.04)*** | .30 | .18 | .35 | .06 | .04 | | .09 |  |
|  |  |  |  | Depression |  | .08  (.01)*** | .47  (.04)*** |  | .24 | .38 | .09 | .06 | | .12 |  |
|  |  |  |  | Stress |  | .06  (.01)*** | .44  (.04)*** |  | .19 | .37 | .07 | .04 | | .09 |  |
| Physical Abuse |  | Anxious  Attachment |  | Anxiety | .92  (.18)*** | .08  (.01)*** | .51  (.06)*** | .16 | .24 | .27 | .07 | .05 | | .11 |  |
|  |  |  |  | Depression |  | .10  (.01)*** | .62  (.06)*** |  | .30 | .32 | .10 | .06 | | .14 |  |
|  |  |  |  | Stress |  | .09  (.01)*** | .43  (.06)*** |  | .26 | .23 | .08 | .05 | | .12 |  |
| Sexual Abuse |  | Anxious  Attachment |  | Anxiety | .54  (.18)** | .09  (.01)*** | .48  (.06)*** | .10 | .26 | .26 | .05 | .02 | | .08 |  |
|  |  |  |  | Depression |  | .11  (.01)*** | .44  (.06)*** |  | .33 | .23 | .06 | .03 | | .10 |  |
|  |  |  |  | Stress |  | .09  (.01)*** | .37  (.05)*** |  | .28 | .21 | .05 | .02 | | .08 |  |
| Emotional Neglect |  | Anxious  Attachment |  | Anxiety | 1.04  (.11)*** | .07  (.01)*** | .30  (.04)*** | .30 | .20 | .26 | .07 | .05 | | .10 |  |
|  |  |  |  | Depression |  | .09  (.01)*** | .42  (.04)*** |  | .24 | .35 | .09 | .06 | | .12 |  |
|  |  |  |  | Stress |  | .07  (.01)*** | .30  (.04) |  | .22 | .26 | .08 | .05 | | .10 |  |
| Physical Neglect |  | Anxious  Attachment |  | Anxiety | 1.08  (.17)*** | .08  (.01)*** | .48  (.05)*** | .21 | .23 | .28 | .08 | .05 | | .11 |  |
|  |  |  |  | Depression |  | .10  (.01)*** | .54  (.05)*** |  | .29 | .30 | .11 | .07 | | .15 |  |
|  |  |  |  | Stress |  | .08  (.01)*** | .37  (.05)*** |  | .25 | .22 | .09 | .06 | | .13 |  |
| Early Life Trauma |  | Avoidant  Attachment |  | Anxiety | .99  (.04)*** | .01  (.01) | .14  (.01)*** | .61 | .06 | .38 | .01 | -.00 | | .03 |  |
|  |  |  |  | Depression |  | .05  (.01)*** | .14  (.01)*** |  | .20 | .36 | .05 | .03 | | .07 |  |
|  |  |  |  | Stress |  | .02  (.01)* | .13  (.01)*** |  | .07 | .36 | .02 | -.00 | | .03 |  |
| Emotional Abuse |  | Avoidant  Attachment |  | Anxiety | 2.56  (.15)*** | .03  (.01)*** | .41  (.04)*** | .49 | .12 | .35 | .07 | .03 | | .11 |  |
|  |  |  |  | Depression |  | .06  (.01)*** | .40  (.04)*** |  | .26 | .32 | .16 | .12 | | .20 |  |
|  |  |  |  | Stress |  | .02  (.01)** | .44  (.04)*** |  | .11 | .38 | .06 | .02 | | .10 |  |
| Physical Abuse |  | Avoidant  Attachment |  | Anxiety | 3.59  (.24)*** | .04  (.01)*** | .42  (.06)*** | .44 | .19 | .22 | .16 | .10 | | .22 |  |
|  |  |  |  | Depression |  | .08  (.01)*** | .44  (.06)*** |  | .32 | .22 | .27 | .21 | | .34 |  |
|  |  |  |  | Stress |  | .05  (.01)*** | .34  (.06)*** |  | .21 | .19 | .17 | .11 | | .23 |  |
| Sexual Abuse |  | Avoidant  Attachment |  | Anxiety | 2.05  (.25)*** | .05  (.01)*** | .42  (.06)*** | .26 | .23 | .23 | .11 | .08 | | .15 |  |
|  |  |  |  | Depression |  | .09  (.01)*** | .32  (.06)*** |  | .37 | .17 | .18 | .14 | | .24 |  |
|  |  |  |  | Stress |  | .06  (.01)*** | .31  (.06)*** |  | .25 | .17 | .11 | .08 | | .16 |  |
| Emotional Neglect |  | Avoidant  Attachment |  | Anxiety | 3.23  (.13)*** | .03  (.01)*** | .27  (.05)*** | .64 | .14 | .23 | .11 | .04 | | .17 |  |
|  |  |  |  | Depression |  | .06  (.01)*** | .31  (.05)*** |  | .25 | .26 | .19 | .13 | | .25 |  |
|  |  |  |  | Stress |  | .03  (.01)*** | .28  (.05)*** |  | .13 | .24 | .10 | .04 | | .16 |  |
| Physical Neglect |  | Avoidant  Attachment |  | Anxiety | 3.95  (.21)*** | .04  (.01)*** | .41  (.06)*** | .52 | .17 | .23 | .15 | .09 | | .22 |  |
|  |  |  |  | Depression |  | .08  (.01)*** | .35  (.06)*** |  | .32 | .19 | .30 | .23 | | .37 |  |
|  |  |  |  | Stress |  | .05  (.01)*** | .28  (.06)*** |  | .21 | .16 | .18 | .11 | | .25 |  |
| **Note**. ****p* < .001; ***p* < .01; **p* < .05; results sequence: ELT - > Mediator; Mediator -> Psychological Symptoms; ELT -> Negative Emotional states | | | | | | | | | | | | | | | |
